# Editorial

**Published:** 2013-09-06

**Authors:** Shibal Bhartiya, Tarek Shaarawy, Tanuj Dada

…neither is it possible to discover the more remote and deeper parts of any science, if you stand but upon the level of the same science, and ascend not to a higher science.

―Sir Francis Bacon

The editorial team of the Journal of Current Glaucoma Practice takes great pleasure in bringing to you this issue of the Journal. We hope you will find this issue insightful and relevant to your practice of glaucoma.

In the epidemiology section, Rajendrababu et al have emphasized on the prevalence of open angle glaucoma screening among first-degree relatives of persons with glaucoma. They also commented that significant barriers exist in the uptake of eye care services among this subgroup.

Bhagat et al commented on the importance of ganglion cell complex (GCC) analysis as a parameter for early diagnosis of glaucoma and for following glaucoma progression and compared it to glaucoma progression as determined with conventional visual field analysis. They report that GCC analysis plays an important role in detecting preperimetric glaucoma and may show progression earlier than pRNFL in preperimetric glaucoma.

Neovascular glaucoma presents a therapeutic challenge for the glaucomatologist. Hernandez-Oteyza et al have reviewed the treatment outcomes of Ahmed glaucoma valve implantation, this disease subgroup, along with risk factors and treatment outcomes. They conclude that a postoperative hypertensive phase and a preoperative BCVA worse or equal to 20/100 seem to be risk factors for surgical failure.

Vieira et al have elucidated and described the technique of deep sclerectomy with the new Esnoper-Clip^®^ implant, the clinical outcome and the anatomic characteristics of filtering blebs, using anterior segment optical coherence tomography. A clinical follow-up of their first five deep sclerectomy with Esnoper-Clip implantation suggests that it may be an effective and well-tolerated method to reduce intraocular pressure, and requires further clinical evalauation.

Kawamorita et al critically analyzed the early postoperative complications in two different tube ligation methods during the first 3 months in Baerveldt implant surgery, presenting evidence that both ligation with absorbable and nonabsorbable methods are effective; however, the selection of tube ligation method should be done in accordance with the different method-specific risks to which may occur.

Hoskens et al have reported the case of a 75-year-old woman who developed an acute bilateral angle closure associated with choroidal effusion a day after an uneventful cataract surgery and suggest that the use of acetazolamide may be a safe and effective treatment of postoperative choroidal effusion and should not be ruled out as a treatment option. As always, we look forward to hearing from you.

Best wishes

**Shibal Bhartiya**

**Tarek Shaarawy**

**Tanuj Dada**

